# Are exergames an option to cope with sleep disorders during the COVID-19 outbreak?

**DOI:** 10.5935/1984-0063.20210030

**Published:** 2022

**Authors:** João Paulo Pereira Rosa, Dayane Ferreira Rodrigues, Ricardo Borges Viana, Rodrigo Luiz Vancini, Marília Santos Andrade, Claudio Andre Barbosa de-Lira

**Affiliations:** 1 Universidade Federal de Goiás, Faculdade de Educação Física e Dança - Goiânia - Goiás - Brazil.; 2 Exersci Scientific Consulting, Sleep and Exercise - Uberlândia - Minas Gerais - Brazil.; 3 Universidade Estadual de Goiás, Escola Superior de Educação Física e Fisioterapia - Goiânia - Goiás - Brazil.; 4 Universidade Federal do Espírito Santo, Centro de Educação Física e Desportos - Vitória - Espírito Santo - Brazil.; 5 Universidade Federal de São Paulo, Departamento de Fisiologia - São Paulo - São Paulo - Brazil.

**Keywords:** Sleep, Exercise, Coronavirus, Video Games

## Abstract

During the COVID-19 pandemic, factors related to the isolation and quarantine period increased psychobiological distress in the general population around the world, increasing anxiety, emotional stress, and depression, as well as worsening of the quality of sleep. Seeking alternatives to provide support for the implementation of some interventions for well-being and health under pandemic conditions, exergames (active video games) seem to be a feasible alternative to keep people physically active and to positively impact sleep health. In this overview article, we discussed the feasibility of exergames as an option to cope with sleep disorders and improve sleep quality during the COVID-19 outbreak through increasing physical exercise and physical fitness levels.

## INTRODUCTION

Coronavirus disease 2019 (COVID-19), which is caused by severe acute respiratory syndrome coronavirus 2 (SARS-CoV-2), has caused an unprecedented health, social, and economic worldwide crisis and infected, and victimized millions of people^1^. The COVID-19 pandemic has produced lockdowns, quarantines, and social distancing and isolation, which has increased health problems, such as stress, depression, anxiety^2-4^, and sleep disturbances^5,6^.

Previous studies have demonstrated that sleep quality of the population has been impaired throughout the pandemic. For example, a study conducted in China with 7,236 participants showed that 18% of them reported poor sleep quality^7^. Salehinejad et al. (2020)^8^ showed that quantitative sleep parameters (e.g., time to go to bed, sleep duration, and get-up time in the morning), sleep quality factors, and circadian rhythms were negatively affected by home quarantine and poor lifestyle due to the COVID-19 pandemic.

Generally, factors related to isolation, quarantine, anxiety, stress, and economic losses and increased levels of physical inactivity could partly explain the impairment of sleep quality in the general population^9^. A review study conducted by Altena et al. (2020)^10^ postulated that sleep disorders might be related to COVID-19 home confinement due to bright light exposure (e.g., smartphone, tablet or laptop) decreasing melatonin release during the night; low levels of physical exercise and social activity (inability to visit friends and family or attend cultural and leisure events); and time pressures of home office, childcare, and household requirements affecting sleep negatively.

Alternatively, physical exercise is recognized as a non-pharmacological tool that positively impacts sleep quality^11^. However, the COVID-19 outbreak imposed quarantine, isolation, and social distancing, as well as the closures of physical exercise facilities and prohibition from circulating among people, which prevented people from performing physical exercise in indoor and/or outdoor environments depending on the region and country^12^. Thus, staying at home to prevent the spread of COVID-19 infection is a new reality, and it is necessary to maintain or increase physical exercise levels to improve or maintain health. In this sense, home-based physical exercise seems to be an effective method to stay active; however, feelings of tiredness, lack of energy, lack of motivation, lack of skills, lack of resources, and fear of injury might be some barriers to people attempting to adopt an active lifestyle^13^. In the current manuscript, we presented exergames (also known as active video games or exergaming) as a possible home-based physical exercise strategy to increase physical exercise levels, mitigating health problems (such as sleep disturbances) due to the COVID-19 outbreak.

### Physical exercise as an intervention to improve sleep quantity and quality

The literature has solid evidence that physical exercise improve sleep quantity and quality^11^. For example, Kelley and Kelley (2017)^14^ conducted a systematic review and meta-analysis that showed the positive impacts of physical exercise on the apnea-hypopnea index, overall sleep quality, global score, subjective sleep, and sleep latency. Briefly, the beneficial short- and long-term effects of physical exercise on sleep architecture may be explained by bidirectional and interactive pathways incorporating circadian rhythm, metabolic, immune, thermoregulatory, vascular, mood, and endocrine effects^15^.

There are some theories to explain the short- and long-term influence of physical exercise on sleep. The thermoregulation theory is grounded on evidence linking the preoptic area of the anterior hypothalamus to sleep regulation and core temperature downregulation^16^. The theories of body restoration and energy conservation are based on the homeostatic mechanisms regulating sleep, of which total sleep time and slow-wave sleep quality increase with energy expenditure^17^. Here, falling asleep is facilitated by two factors: physiological strains and decrease in body energy caused by physical training, which increases the need for rest and consequently sleep^18^. For this reason, regular physical exercise might promote relaxation and energy expenditure that benefits sleep^19^. Also, regular physical exercise has been associated with improved variables related to sleep, including sleep quality, sleep latency, wake time after sleep onset, and sleep disturbances^20^. By contrast, increased sedentary behavior has been associated with worse sleep outcomes, such as insomnia, sleep disturbances, and insufficient sleep^21^. Therefore, the scientific literature brings evidence that supports the benefits of regular physical exercise as a non-pharmacologic strategy to improve sleep quantity and quality^11^.

In this sense, preventive measures adopted to mitigate COVID-19 transmission included the shutting down of physical exercise facilities (e.g., gyms, swimming pools, and fitness centers) and restriction of outdoor and indoor activities, which may increase physical inactivity and sedentary behavior^22^. To cope with this situation, previous studies have proposed home-based physical exercise programs through feasible and alternative strategies, such as practicing kettlebell training^23^ or interval training^24^ (e.g., high intensity interval training: 1-4 minute bouts of vigorous exercise interspersed with periods of passive or active recovery). However, for some persons, especially for those that are not experienced with physical exercise training, these strategies could not be easy to implement, since there is a need to control the variables related to training, such as intensity and/or guidance from a specialized professional^25^. Thus, a feasible and possible alternative could be exergames.

### Exergames as a strategy to increase levels of physical exercise and quality of sleep during the COVID-19 outbreak

Nowadays, new ways of technology are being created as options for health promotion. Among these options, traditional video games are one of the most popular forms of leisure time^26^. In addition, initiatives in social media (e.g., #PlayApartTogether) promote playing video games for socialization and stress reduction during the COVID-19 pandemic. However, a great amount of time is spent playing video games via online streaming in consoles, which stimulates sedentary behavior^27^. Technology has been looked upon with a certain villainy and has been responsible for declining levels of physical exercise among people around the world^28^. Unlike traditional video games, exergames stimulate the player by moving their whole body, resulting in higher motor activation, and responses of physiological variables (e.g., heart rate, oxygen uptake and energy expenditure) as compared to traditional video games that use joysticks^29^.

Many previous studies have shown that the most common exergames elicit a physical exercise intensity corresponding from light to moderate^29-33^. For example, Viana et al. (2018)^29^ investigated the responses of heart rate and oxygen uptake during an exergame session. The authors found that exergames can be classified as light to moderate exercise and concluded that exergames could be an interesting alternative to traditional forms of physical exercise. Notably, these authors investigated an exergame that involved callisthenic physical exercise, which is a traditional mode of physical exercise performed by physical exercise practitioners and prescribed by coaches. Viana et al. (2017)^32^ and Morais et al. (2021)^34^ investigated the acute effects of the exergame Zumba Fitness on anxiety in women. The authors found that this exergame had an anxiolytic effect and attributed this effect to the moderate intensity of physical exercise evoked by the exergame session.

Given that most studies that have investigated the effect of physical exercise on sleep have applied traditional forms of moderate-intensity physical exercise^35^, it is reasonable to assume that exergames can evoke effects similar to traditional forms of physical exercise on sleep and may be used to manage sleep disorders, especially among those that are physically inactive and that are not attracted to performing traditional forms of physical exercise.

Indeed, the main advantage of exergames compared with traditional forms of physical exercise is that exergames are considered more enjoyable^30,33^. In practical terms, this finding is relevant because more than a quarter of all adults around the world are physically inactive^36^, and one of the barriers to becoming physically inactive pointed out by people is “finding physical activity unattractive.” Therefore, exergames present the potential to attract inactive people to a physical exercise program.

To the best of our knowledge, only one study has investigated the effect of exergames on sleep. Yunus et al. (2020)^37^ investigated the effects of the exergame Kinect Sports for Xbox 360 Kinect on sleep and emotional well-being among students. The participants were submitted to 30 minutes of intervention, three times per week for 6 weeks. The authors found improvement in sleep using the Functional Outcome Sleep Questionnaire-30 in the exergame group but not in the control group. The authors concluded that exergames are feasible to manage sleep conditions.

### Limitations of exergames use and practical recommendations during the COVID-19 outbreak

Some limitations and practical recommendations must be considered about the feasibility of using exergames to cope with sleep disturbances during the COVID-19 outbreak. First, this intervention (exergames) implies the use of a specific console and accessories (to motion capture and control) connected to a TV^38^. These electronic devices may not be accessible or available for everyone, especially those living in low- and middle-income wage brackets, as the costs of the equipment might be prohibitive for some people. Finally, exergames can be difficult to use among older people or those who are not familiar with recent technology. In these cases, more traditional home-based physical exercise programs should be encouraged.

As mentioned above, exergames are considered more fun than traditional forms of physical exercise and a potential tool to increase adherence to regular physical exercise^39^. Considering sustaining motivation to continue playing exergames, different games might be used in a single or multiplayer mode^40^. Thus, multiplayer/single player preferences can increase the level of game enjoyment^40^.

Albeit speculative, it is reasonable to assume that it would be important to select games that induce moderate intensity levels. Considering that the literature does not provide an ideal duration of an exergame session and that exergames elicit a physical exercise intensity similar to that elicited by traditional forms of physical exercise, we recommended to remain active during the COVID-19 outbreak following the World Health Organization 2020 guidelines on physical activity and sedentary behavior (e.g., 150-300 minutes of moderate-intensity aerobic physical activity or 75-150 minutes of vigorous-intensity activity per week)^41^.

The time of day/night which exergames will be undertaken should be considered because late-night physical exercise results in a body temperature increase, increase in blood pressure and heart rate levels, and sympathetic nervous system activation, all of which impair sleep quality^42^. Other studies conducted within non-athletic participants have not observed any adverse effects or disturbed nocturnal sleep^43^ after the physical exercise session, which may be involved with improving sleep quality when performed 2-4 hours before bedtime^17,44^. On the other hand, disturbed nocturnal sleep was observed after high-intensity physical exercise performed 1 hour before bedtime^45^.

The ratio between physical exercise timing and sleep shall be modulated by chronotype. The chronotype is individual and reflects circadian variations of physiological and psychological variables during the day^46^, and this human predisposition to perform better physical or cognitive tasks during morningness or eveningness is supposedly determined by the central circadian clock located in the suprachiasmatic nuclei of the hypothalamus^47^. Considering that there is no consensus about physical exercise timing and sleep, chronotype status might be used to identify the ideal period for exercise based on innate circadian rhythmicity^46^, using self-assessment questionnaires, such as the morningness-eveningness questionnaire^48^.

Another limitation is that the blue light emission of a TV can disrupt the natural sleep-wake cycles of the brain^49^, especially when people engage in exergames before bedtime. However, some practical recommendations to minimize the effects of blue light exposure during an exergame session is the player use amber-tinted glasses to avoid excessive blue light exposure at night^50^ in order to cancel out the melatonin-suppressing effect of the bright light^51^ and change some settings manually on the TV to reduce brightness and contrast or turn on a blue light filter to mitigate sleep disturbances due to excessive or inadequate lighting.

## CONCLUSION

Exergames can be an interesting alternative and attractive tool in times of the COVID-19 pandemic, with lockdowns, quarantines, and social distancing and isolation, to cope with sleep disturbances. In addition, the benefits of the practice of exergames could be innumerable, mainly for populations at risk of getting coronavirus infections, such as children who are still unable to attend school and do group physical exercise programs safely and elderly people who, in general, had lower levels of physical activity, even before the pandemic, and in many cases, already live in a situation of social isolation. Finally, exergames can be played at home with relative safety and could help improve and maintain physical exercise levels, as well as improve the quality of sleep.
